# Burden of influenza hospitalization among high-risk groups in the United States

**DOI:** 10.1186/s12913-022-08586-y

**Published:** 2022-09-28

**Authors:** Aimee M. Near, Jenny Tse, Yinong Young-Xu, David K. Hong, Carolina M. Reyes

**Affiliations:** 1 IQVIA, 4820 Emperor Blvd, Durham, NC 27703 USA; 2 US Department of Veterans Affairs, Clinical Epidemiology Program, 215 N Main St, White River Junction, VT 05009 USA; 3 VIR Biotechnology Inc, 499 Illinois St, San Francisco, CA USA

**Keywords:** Comorbidities, Health resource utilization, Hospitalization, Influenza, Real-world

## Abstract

**Background:**

Seasonal influenza poses a substantial clinical and economic burden in the United States and vulnerable populations, including the elderly and those with comorbidities, are at elevated risk for influenza-related medical complications.

**Methods:**

We conducted a retrospective cohort study using the IQVIA PharMetrics® Plus claims database in two stages. In Stage 1, we identified patients with evidence of medically-attended influenza during influenza seasons from October 1, 2014 to May 31, 2018 (latest available data for Stage 1) and used a multivariable logistic regression model to identify patient characteristics that predicted 30-day influenza-related hospitalization. The findings from Stage 1 informed high-risk subgroups of interest for Stage 2, where we selected cohorts of influenza patients during influenza seasons from October 1, 2014 to March 1, 2019 and used 1:1 propensity score matching to patients without influenza with similar high-risk characteristics to compare influenza-attributable rates of all-cause hospital and emergency department (ED) visits during follow-up (30-day and in the index influenza season).

**Results:**

In Stage 1, more than 1.6 million influenza cases were identified, of which 18,509 (1.2%) had a hospitalization. Elderly age was associated with 9 times the odds of hospitalization (≥65 years vs. 5–17 years; OR = 9.4, 95% CI 8.8–10.1) and select comorbidities were associated with 2–3 times the odds of hospitalization. In Stage 2, elderly influenza patients with comorbidities had 3 to 7 times higher 30-day hospitalization rates compared to matched patients without influenza, including patients with congestive heart failure (41.0% vs.7.9%), chronic obstructive pulmonary disease (34.6% vs. 6.1%), coronary artery disease (22.8% vs. 3.8%), and late-stage chronic kidney disease (44.1% vs. 13.1%; all *p* < 0.05).

**Conclusions:**

The risk of influenza-related complications is elevated in the elderly, especially those with certain underlying comorbidities, leading to excess healthcare resource utilization. Continued efforts, beyond currently available vaccines, are needed to reduce influenza burden in high-risk populations.

**Supplementary Information:**

The online version contains supplementary material available at 10.1186/s12913-022-08586-y.

## Background

Despite the availability of vaccines, the burden of seasonal influenza remains high in the United States (US), contributing to excess morbidity, mortality, and healthcare resource utilization (HRU). The Centers for Disease Control and Prevention (CDC) estimates that influenza accounted for 4.3–21 million medical visits, 140,000–810,000 hospitalizations, and 12,000–61,000 deaths annually in the US during the 2010–11 through 2019–20 influenza seasons [1]. In turn, the estimated total economic burden of influenza is substantial at $11.2 billion (ranging from $6.3–$25.3 billion) [2] and as high as $87.1 billion (95% confidence interval [CI], $47.2–$149.5) [3]. Direct medical costs have been estimated at $3.2 billion annually, of which 70% ($2.3 billion) is due to hospitalizations [2], despite hospitalization in only 1–2% of medically-attended influenza cases [1].

Although influenza is generally self-limiting with mild symptoms in healthy individuals [4], certain vulnerable populations are at elevated risk for serious influenza-related medical complications. For example, while the elderly population ≥ 65 years of age has the lowest median incidence of influenza (3.9%) compared to children 0–17 years (9.3%) or adults 18–64 years (8.8%) [5], they account for 50–70% of influenza-related hospitalizations, 70–85% of deaths [6], and 42.7% of direct medical costs [2]. Chronic medical conditions, including pulmonary, cardiovascular, renal, hepatic, and metabolic disorders, have also been identified as predictors of influenza-related complications [7–12]. Vaccination is recognized as the most effective prevention strategy for seasonal influenza, but in the 2019–2020 season, only 51.8% of persons 6 months or older were vaccinated [13].

As both vaccination against influenza infections and treatment for complications evolve, there is limited up-to-date research quantifying risk factors associated with hospitalization or the added healthcare resource burden attributable to seasonal influenza among patients vulnerable to complications using real-world data. To that end, this study aimed to identify risk factors for influenza-related hospitalization (Stage 1) and to evaluate the burden of influenza in at-risk elderly populations (Stage 2).

## Methods

### Study data source

The data source for this retrospective cohort study was IQVIA PharMetrics® Plus, a health plan claims database comprised of fully adjudicated medical and pharmacy claims from more than 100 commercial health plans, covering more than 150 million unique enrollees representative of the commercially insured US population (see Supplemental Fig. 1) [14, 15]. All data are Health Insurance Portability and Accountability Act (HIPAA)-compliant to protect patient privacy [16, 17]. As this retrospective cohort analysis was conducted using de-identified HIPAA-compliant data, Institutional Review Board (IRB) review was not required for this study.

### Study period and population

This study was conducted in two stages, with separate patient selection criteria in each stage. In Stage 1, a cohort of patients of any age with evidence of ≥1 influenza diagnosis from an inpatient claim or non-ancillary outpatient claim (i.e. “medically-attended influenza patients”) were identified in the database from October 1, 2014 to May 31, 2018, a timeframe that includes the four most recent influenza seasons at the time of data extraction. The index date was the date of the earliest observed influenza diagnosis occurring during an influenza season (spanning from October 1 to May 31 of the subsequent year). Patients were required to have ≥12 months of continuous enrollment in their health plan before index (baseline) and ≥ 30 days after index (follow-up) and either index influenza diagnosis in the primary position or evidence of influenza lab test order ±14 days of index. Influenza diagnoses were identified using International Classification of Diseases, 9th and 10th revision (ICD-9/10) codes (ICD-9: 487–488; ICD-10: J09-J11). Influenza patients who met all inclusion and exclusion criteria were stratified into two mutually exclusive groups (hospitalized vs. non-hospitalized) based on the presence of ≥1 influenza-related hospitalization. Influenza-related hospitalization was defined as an inpatient visit with a diagnosis code for influenza or an influenza-related complication (respiratory, renal, cardiovascular, neurological/musculoskeletal, and other conditions [e.g., conjunctivitis, dehydration, liver inflammation/hepatitis, sepsis]) associated with the claim in any position within 30 days after the index influenza diagnosis [18–23].

In Stage 2, patients who were ≥ 65 years of age were identified during the study period from October 1, 2013 to March 31, 2019 and then selection criteria were applied separately to the influenza cohort and the non-influenza cohort. Selection criteria for the influenza cohort were similar to those applied in Stage 1 and the index date was the date of the earliest influenza diagnosis occurring during an influenza season from October 1, 2014 to March 1, 2019. Patients in the non-influenza comparator cohort had no evidence of influenza diagnosis during the study period and their index dates were assigned to replicate the distribution of index dates observed in the influenza cohort. The elderly influenza and non-influenza cohorts were further stratified into 12 non-mutually exclusive high-risk subgroups (broadly categorized as pulmonary disease, cardiovascular disease, or renal disease) characterized by baseline comorbidities. Patients were required to have evidence of ≥1 inpatient or ≥ 2 outpatient diagnoses during the 12-month baseline period for any of the following high-risk subgroups: asthma, chronic obstructive pulmonary disease (COPD), chronic pulmonary disease, atherosclerosis, coronary artery disease (CAD), congestive heart failure (CHF), stroke, valvular disease, old myocardial infarction (MI; defined using diagnosis codes indicating history of MI), acute MI, late stage chronic kidney disease (CKD; including CKD stage 5, end-stage renal disease, or dialysis), and early stage CKD (including CKD stages 3 or 4). These subgroups were selected based on results from Stage 1 analyses [24], which investigated 29 comorbidities from the Agency for Healthcare Research and Quality (AHRQ) comorbidity software [25] and 10 chronic medical conditions identified by the CDC as increasing the risk for serious influenza-related complications [26]. The corresponding diagnosis codes can be found in Supplementary file 1.

Within each of the 12 high-risk subgroups, the influenza and non-influenza cohorts were matched using propensity score (PS) matching at a 1:1 ratio with a greedy nearest-neighbor matching algorithm, without replacement and using caliper widths of 0.1 of the standard deviation (SD) of the logit of the PS. The logistic regression model to generate the PS included patient demographics, baseline clinical characteristics, and baseline all-cause healthcare costs (USD; detailed list of variables can be found in Supplementary Table 2 footnotes and are listed in Supplementary Tables 3, 4, 5, 6, 7, 8, 9, 10, 11, 12, 13 and 14).

### Study measures and statistical analysis

#### Stage 1

Patient demographic and baseline clinical characteristics were assessed during the 12-month baseline period. Comorbidities of interest were derived from 29 comorbidities from the AHRQ comorbidity software [25] and 10 chronic medical conditions identified by the CDC as increasing the risk for serious influenza-related complications [26]. Influenza vaccination was defined as ≥1 National Drug Code (NDC) or Healthcare Common Procedure Coding System (HCPCS) code for influenza vaccination occurring during the index influenza season and prior to the index date. Among patients with evidence of influenza-related hospitalization, the total cost of the hospitalization, which includes the cost paid by the health plan to the service provider and any patient out-of-pocket costs, was also reported.

All measures were reported as descriptive statistics by group defined by influenza hospitalization status. Statistical differences between the groups were assessed using a two-sided t-test comparing means of continuous variables and Pearson’s chi-square test for categorical variables. A logistic regression model was used to estimate odds ratios (ORs) and 95% CIs for the association between patient demographic and baseline clinical characteristics and the odds of influenza-related hospitalization.

#### Stage 2

Patient demographic and baseline clinical characteristics were assessed during the 12-month baseline period. Follow-up measures were evaluated in the matched cohorts, including all-cause hospitalizations and emergency department (ED) visits within 30 days after the index date and during the index influenza season.

Baseline patient characteristics between the influenza and non-influenza cohorts before and after PS matching were assessed using standardized difference, applying a commonly used threshold of > 10% in absolute standardized difference to determine imbalance [27]. Follow-up measures were compared using McNemar’s Chi-squared test for paired data. *P*-values < 0.05 were considered statistically significant. Analyses were conducted using SAS version 9.4 (SAS Institute, Inc., Cary, NC).

## Results

### Stage 1 results

#### Study population

**Fig. 1:**
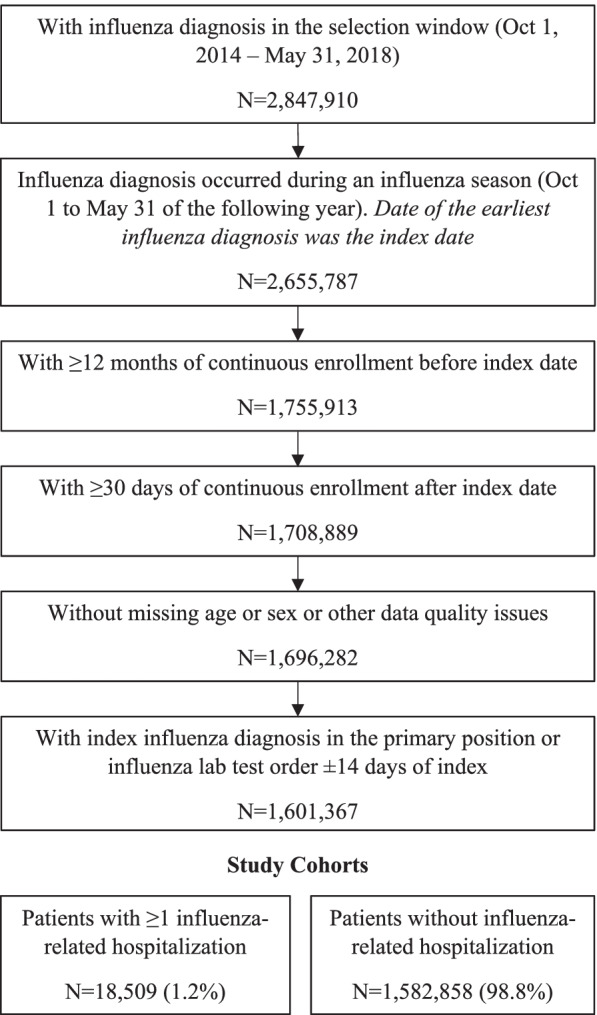
Stage 1 patient selection criteria. Influenza was identified using ICD-9 codes 487–488 and ICD-10 codes J09-J11

In total, 1,601,367 medically-attended influenza patients met the study inclusion criteria (Fig. 1). Of these, 18,509 (1.2%) patients had ≥1 influenza-related hospitalization within 30 days after the index date and 1,582,858 (98.8%) patients did not have evidence of hospitalization.

#### Patient demographic and baseline characteristics

Medically-attended influenza patients who were hospitalized generally differed from those not hospitalized, in terms of baseline demographic and clinical characteristics (Table 1).

**Table 1:** Baseline demographic and clinical characteristics by hospitalization status

Baseline characteristics	Patients with ≥1 influenza-related hospitalization *N* = 18,509	Patients without influenza-related hospitalization *N* = 1,582,858	*p*-value
Age, mean ± SD	47.3 ± 23.1	28.8 ± 19.8	< 0.0001
Age group, n (%)			< 0.0001
0–4	1201 (6.5)	125,906 (8.0)	
5–17	1807 (9.8)	512,517 (32.4)	
18–49	4926 (26.6)	623,281 (39.4)	
50–64	6787 (36.7)	284,492 (18.0)	
65+	3788 (20.5)	36,662 (2.3)	
Sex, n (%)			0.71
Male	8749 (47.3)	745,993 (47.1)	
Female	9760 (52.7)	836,865 (52.0)	
Payer type, n (%)			< 0.0001
Commercial	9374 (50.7)	866,656 (54.8)	
Self-insured	6426 (34.7)	599,540 (37.9)	
Medicaid	1769 (9.6)	108,617 (6.9)	
Medicare risk	852 (4.6)	2509 (0.2)	
Other/Unknown	88 (0.5)	5536 (0.3)	
Geographic region, n (%)			< 0.0001
Northeast	3457 (18.7)	188,490 (11.9)	
Midwest	4758 (25.7)	334,387 (21.1)	
South	7583 (41.0)	906,543 (57.3)	
West	2711 (14.7)	153,438 (9.7)	
AHRQ/CDC comorbidities^a^, mean ± SD	3.8 ± 3.5	1.0 ± 1.6	< 0.0001
AHRQ/CDC comorbidities category^a^, n (%)			< 0.0001
0	3510 (19.0)	891,422 (56.3)	
1–3	6924 (37.4)	569,667 (36.0)	
4–6	4456 (24.1)	102,322 (6.5)	
7–9	2221 (12.0)	15,774 (1.0)	
10+	1396 (7.5)	3421 (0.2)	
Index influenza season, n (%)			< 0.0001
2014	4725 (25.5)	444,002 (28.1)	
2015	3533 (19.1)	230,985 (14.6)	
2016	4373 (23.6)	379,894 (24.0)	
2017	5878 (31.8)	527,977 (33.4)	
Evidence of influenza vaccination before influenza diagnosis in the index season^a^, n (%)	3954 (21.4)	280,898 (17.7)	< 0.0001

*AHRQ* Agency for Health Research and Quality, *CDC* Centers for Disease Control and Prevention, *SD* Standard deviation, *SD* Standard deviation

^a^Patients could have a maximum of 37 comorbidities defined using ICD-9/ICD-10 codes from the Elixhauser Comorbidity Software (AHRQ) and groups defined by the CDC as at high-risk for influenza-related hospitalizations

**Fig. 2:**
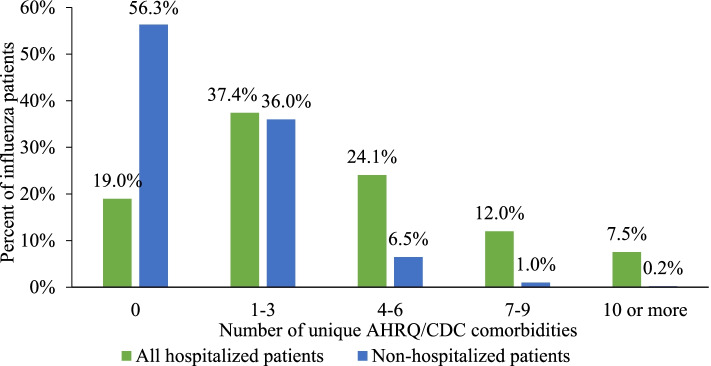
Number of baseline AHRQ/CDC comorbidities by influenza-related hospitalization status. AHRQ: Agency for Health Research and Quality, CDC: Centers for Disease Control and Prevention. *Patients could have a maximum of 37 comorbidities defined using ICD-9/ICD-10 codes from the Elixhauser Comorbidity Software (AHRQ) and groups defined by the CDC as at high-risk for influenza-related hospitalizations

Hospitalized patients were, on average, nearly 20 years older compared to non-hospitalized patients (mean age, 47.3 vs. 28.8 years; *p* < 0.0001) and were more likely to be ≥65 years of age (20.5% vs. 2.3%). In addition, a larger proportion of hospitalized patients had Medicaid (9.6% vs. 6.9%) or Medicare Risk (4.6% vs. 0.2%) plans compared to non-hospitalized patients (*p* < 0.0001). Hospitalized patients also had a higher baseline comorbidity burden (mean AHRQ/CDC comorbidities, 3.8 vs. 1.0, *p* < 0.0001) and 43.6% of hospitalized patients had 4+ comorbidities compared to 7.7% of non-hospitalized patients (Fig. 2).

**Fig. 3:**
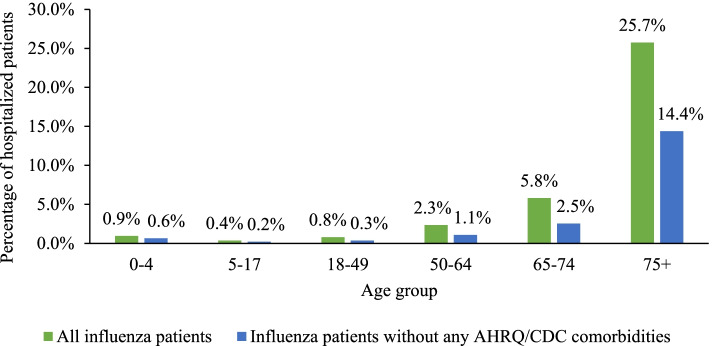
Influenza-related hospitalization rates of patients without any AHRQ/CDC comorbidities in the baseline period compared to all influenza patients. AHRQ: Agency for Health Research and Quality, CDC: Centers for Disease Control and Prevention

Furthermore, in the entire sample of influenza patients, 5.8% of patients ages 65–74 and 25.7% of patients ages ≥75 were hospitalized within 30 days after the index date, compared to 2.3, 0.8, 0.4, and 0.9% of patients ages 50–64, 18–49, 5–17, and 0–4, respectively. These age trends persisted among patients without evidence of any AHRQ/CDC comorbidities during baseline (Fig. 3).

Lastly, 21.4% of hospitalized patients had evidence of an influenza vaccine during the respective influenza season in the claims database compared to 17.7% of non-hospitalized patients (*p* < 0.0001).

#### Predictors of influenza-related hospitalization

**Fig. 4:**
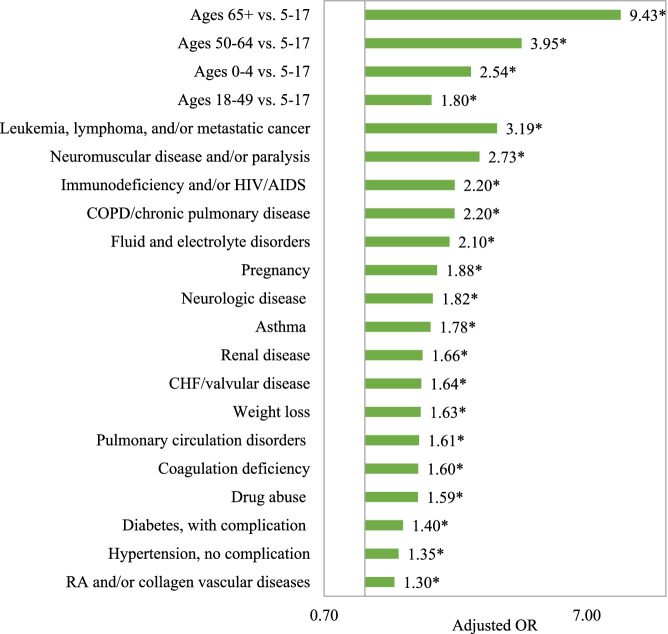
Logistic regression model for the odds of influenza-related hospitalization, adjusting for baseline demographic and clinical characteristics. CHF: Congestive heart failure, COPD: Chronic obstructive pulmonary disease, HIV/AIDS: Human Immunodeficiency Virus/Acquired Immunodeficiency Syndrome, RA: Rheumatoid arthritis. *Indicates significant difference between hospitalized and non-hospitalized patients (*p* < 0.05). Logistic regression model adjusted for age, sex, payer type, geographic region, index influenza season, evidence of influenza vaccination, and specific AHRQ/CDC comorbidities. This figure shows the odds ratios only for age groups and for select comorbidities where the odds ratio was > 1.30. Other comorbidities significantly associated with the odds of influenza-related hospitalization included rheumatoid arthritis/collagen vascular diseases, diabetes with no complication, deficiency anemias, solid tumor without metastasis, alcohol abuse, hypertension with complication, peripheral vascular disorder, liver disease, psychoses, and depression (odds ratios ranging from 1.08 to 1.30). Blood loss anemia, obesity, other metabolic disease, hypothyroidism, and chronic peptic ulcer disease were not associated with the odds of influenza-related hospitalization

Elderly age and specific comorbidities were identified as predictors of influenza-related hospitalization in the logistic regression model, adjusted for baseline demographic and clinical characteristics (Fig. 4).

Specifically, elderly patients (≥65 years) had 9.4 times higher odds of hospitalization compared to patients aged 5–17 years (95% CI, 8.8–10.1). Patients with certain groupings of comorbidities during baseline also had 2–3 times the odds of influenza-related hospitalization compared to patients without those underlying conditions, including leukemia, lymphoma and/or metastatic cancer; neuromuscular disease and/or paralysis, immunodeficiency and/or Human Immunodeficiency Virus/Acquired Immunodeficiency Syndrome [HIV/AIDS]; and COPD and/or chronic pulmonary disease) (all *p* < 0.0001). Full model results can be found in Supplementary Table 1.

### Stage 2 results

#### Study population

**Fig. 5:**
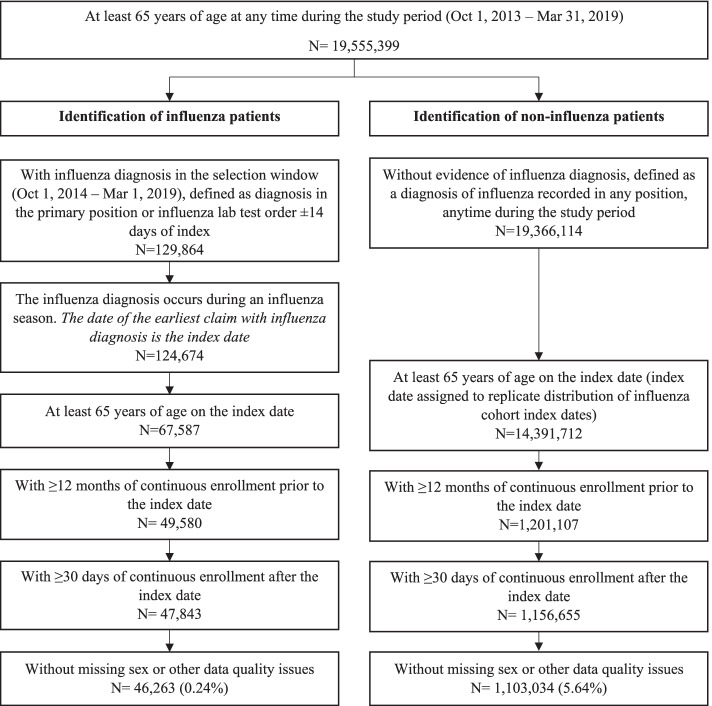
Stage 2 patient selection criteria

Given the higher odds of influenza-related hospitalization observed among elderly patients in Stage 1, we identified a sample of influenza and non-influenza patients ages ≥65 years from October 1, 2013 – March 31, 2019. In total, 46,263 influenza and 1,103,034 non-influenza patients met study selection criteria (Fig. 5) and were further categorized into 12 influenza and 12 non-influenza cohorts with ≥1 comorbidity of interest.

Patient demographic and baseline clinical characteristics and baseline total all-cause healthcare costs were generally well-balanced after 1:1 PS-matching based on the threshold of standardized difference > 10% to define imbalance After matching, some imbalances remained (standardized differences up to 15%) for the acute MI and old MI, atherosclerosis, chronic pulmonary disease and late stage CKD cohorts for payer type, plan type, and timing of the index date (index season and month). Cohort sizes and characteristics after PS-matching are provided in Supplementary Tables 3, 4, 5, 6, 7, 8, 9, 10, 11, 12, 13, 14.

#### All-cause hospitalization and ED visits during follow-up

**Fig. 6:**
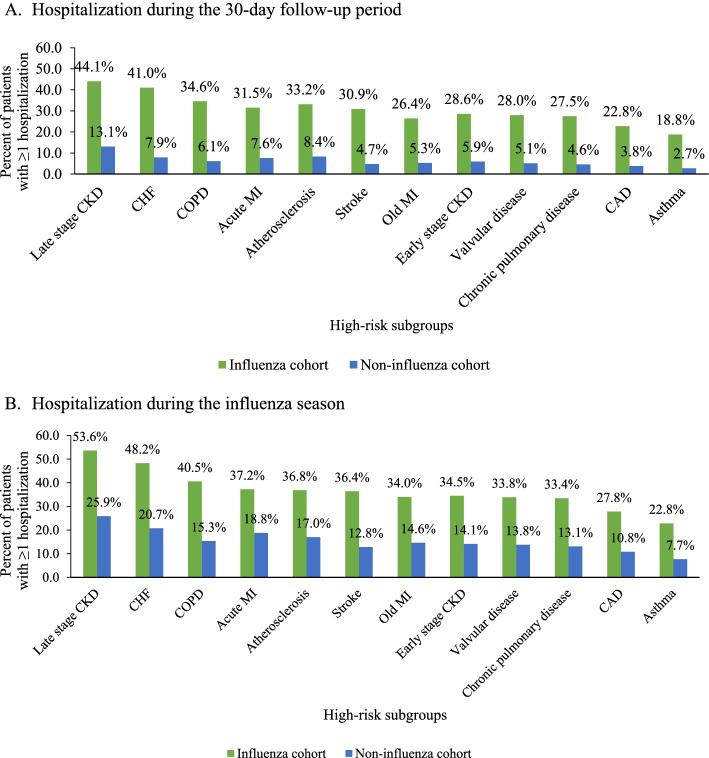
All-cause hospitalization rates in the elderly influenza and non-influenza cohorts by comorbidity status. **A** Hospitalization during the 30-day follow-up period. **B** Hospitalization during the influenza season. CAD: Coronary artery disease, CHF: Congestive heart failure, CKD: Chronic kidney disease, COPD: Chronic obstructive pulmonary disease, MI: Myocardial infarction. All *p*-values for comparisons of hospitalization rates between the influenza and non-influenza cohorts < 0.05

Overall, the frequency of ≥1 all-cause hospitalization was higher for the influenza cohort during the 30-day follow-up period and during the index influenza season compared to matched controls for all comorbidity subgroups. During the 30-day follow-up period, the influenza cohorts had 3–7 times higher rates of hospitalization compared to the corresponding non-influenza cohorts; these differences were most prominent for CHF (41.0% vs. 7.9%), COPD (34.6% vs. 6.1%), CAD (22.8% vs. 3.8%), and late stage CKD (44.1% vs. 13.1%; all *p* < 0.0001) (Fig. 6a). The differences were smaller when broadening follow-up to the influenza season, with 2–3 times higher hospitalization rates for the influenza cohorts compared to the matched non-influenza cohorts: CHF (48.2% vs. 20.7%), COPD (40.5% vs. 15.3%), CAD (27.8% vs. 10.8%), or late stage CKD (53.6% vs. 25.9%; all *p* < 0.0001) (Fig. 6b). In Stage 1, the mean total cost of influenza-related hospitalization among the hospitalized cohort was $22,169 (SD, $61,593) and the median cost was $11,014.8 (quartile 1, $6653; quartile 3; $19,246).

**Fig. 7:**
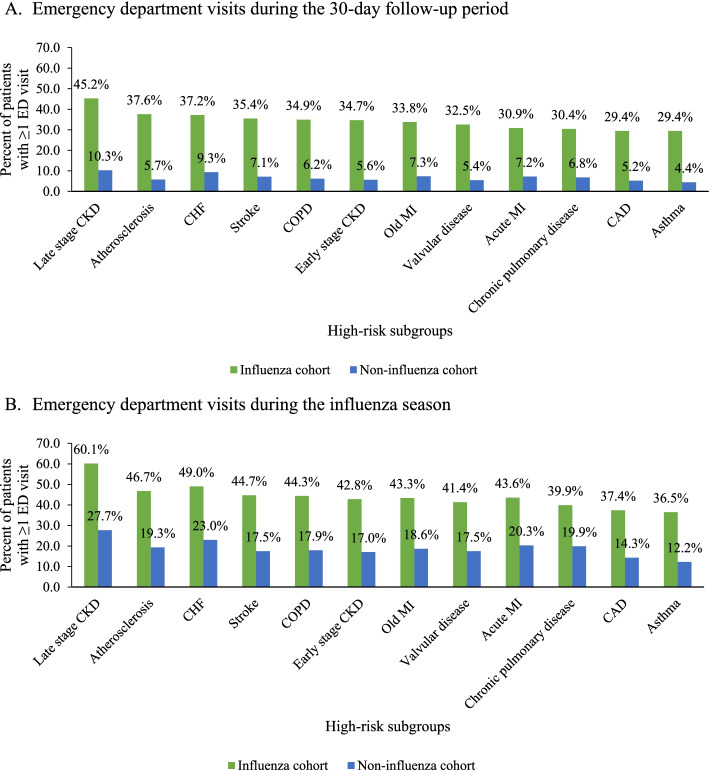
Emergency department visits during the 30-day follow-up period in the elderly influenza and non-influenza cohorts by comorbidity status. **A** Emergency department visits during the 30-day follow-up period. **B** Emergency department visits during the influenza season. CAD: Coronary artery disease, CHF: Congestive heart failure, CKD: Chronic kidney disease, COPD: Chronic obstructive pulmonary disease, ED: Emergency department, MI: Myocardial infarction. All *p*-values for comparisons of ED visit rates between the influenza and non-influenza cohorts < 0.05

Similar findings were also observed for ED visits. During the 30-day follow-up period, influenza patients with CHF, COPD, CAD, or late stage CKD had 4–6 times higher ED visit rates compared to the corresponding non-influenza cohort (37.2% vs. 9.3, 34.9% vs. 6.2, 29.4% vs. 5.2, 45.2% vs. 10.3%, respectively; all *p* < 0.0001) (Fig. 7a). During the influenza season, ED visit rates were about two times higher in influenza patients compared to non-influenza patients; all *p* < 0.0001) (Fig. 7b).

## Discussion

The findings of this real-world study demonstrate that the elderly population and those with specific comorbidities are at elevated risk of influenza-related complications that may result in hospitalizations, a primary driver of the substantial healthcare resource burden of the disease. While the severity of influenza can vary with every season, the susceptibility of the elderly to disease-related complications has been consistently reported in epidemiological studies across geographies and has been attributed to alterations in immune defenses with age [28]. In our study, we identified more than 1.6 million influenza patients. Of these patients, only 2.5% were elderly and yet they accounted for 20.5% of influenza-related hospitalizations. Apart from age-related vulnerability, our study also identified that certain pre-existing comorbidities have a direct impact on outcomes, increasing the odds of hospitalization by 2 to 3-fold. Similar findings were reported in a meta-analysis of populations at risk for severe or complicated seasonal influenza, which found that the elderly had higher risk of hospitalization and death compared to the non-elderly (OR, 3.0; 95% CI, 1.5–5.7). Additionally, comorbidities were strongly associated with death (OR, 2.0; 95% CI, 1.7–2.4) and conditions like chronic lung disease, cardiovascular disease, COPD, and diabetes increased the probability of hospitalization and, in some cases, ventilator support [10].

Seasonal influenza-related complications and hospitalizations are not only life-threatening to populations at risk, but also a substantial economic burden for the healthcare system [2] and persistent efforts are needed to alleviate the burden in the high-risk populations highlighted in this study through vaccinations, early treatment, and existing prophylactic medications [23] or novel targeted therapies, such as monoclonal antibodies [29]. Currently, vaccination is the most promising method to prevent and control influenza by reducing the risk of ailment by 40–60% and reducing disease severity among patients with post-vaccination infections [30]. Although the US vaccination target of 70% [31] was not achieved in the 2019–20 influenza season, with only 51.8% of the population aged ≥6 months receiving a vaccination [32], the impact of vaccination on morbidity and mortality was sizeable. Using a model accounting for vaccination coverage, vaccine efficacy, and disease occurrence, the CDC estimated that vaccinations prevented 7.5 million influenza illnesses, 3.7 million influenza-associated medical visits, 105,000 influenza-associated hospitalizations, and 6300 influenza-associated deaths in the 2019–20 season [33, 34].

Achieving the vaccination targets for influenza would allow for even larger impacts, but complacency and concerns regarding safety and efficacy are factors responsible for lack of widespread vaccination [35, 36]. For the elderly population at risk for complications, the vaccination target in the US is even higher at 90%, yet this target is rarely reached in the industrialized world [37]. However, the effectiveness of the vaccine in the elderly is known to be reduced due to lower seroconversion rates from poorer immunologic response [38] and in Stage 1 of this study, we observed higher rates of vaccination among influenza patients with hospitalization (21.4%) compared to patients without hospitalization (17.7%). Hence, despite the efforts towards widespread vaccination programs, there remains an unmet need for preventing complications of influenza in vulnerable populations, even among those who received vaccinations.

The strength of our study lies in estimating influenza-related hospitalizations in the backdrop of risk factors like pre-existing comorbidities, thus adding to the current literature related to influenza-related HRU using the most recently available data at the time of the study. The existing CDC model for reporting influenza-associated hospitalizations uses the reported number of hospitalizations to calculate hospitalization rates, which are adjusted to compensate for any under-detection. The adjusted rates are then applied to the US population by age group to estimate total number of influenza-related hospitalizations [39]. This method is limited due to lack of inclusion of the full denominator of patient groups at-risk for influenza because of underlying comorbid conditions, which can result in inadequacies when assessing the actual healthcare resource burden.

Several limitations of this retrospective database analysis must be noted. First, clinical data were not available in this study, which may have led to misclassification of the influenza diagnoses due to the inability to verify disease status with test results. To account for this, we required evidence of an influenza lab test within 14 days of influenza diagnosis (in any position) or primary diagnosis of influenza on the claim. This study likely also underestimates the receipt of influenza vaccinations prior to the index date since we observed only about one-third of patients across the influenza and non-influenza cohorts with documented vaccination in the claims database. Only vaccinations documented by insurance would be captured in the study database; free vaccines or vaccines paid by cash are not captured. Secondly, we did not investigate outcomes like rehabilitation and nursing home care after hospitalization, which contribute to the long-term disease burden not captured in the present study and should be evaluated in future real-world studies using longer follow-up periods. Thirdly, comparisons of outcomes between the influenza and non-influenza cohorts stratified by influenza season would have been valuable in understanding the impact of influenza subtype/lineage on healthcare resource burden. Although stratified analyses were not conducted and laboratory data were not available, this study nevertheless provides valuable insights summarized over multiple influenza seasons. Fourthly, our comparisons of hospitalizations and ED visits between the influenza and non-influenza cohorts may be biased due to residual confounding from matching factors that remained slightly imbalanced after matching and unmeasured sociodemographic characteristics that may be associated with healthcare resource utilization (e.g., race/ethnicity and income). In addition, data for the end of the 2018–2019 season were not available at the time this study was conducted, which may have impacted findings in Stage 2; however, we anticipate any bias would be limited since the timing of the index date (index season and month) were considered in matching the influenza and non-influenza cohorts. Lastly, given the study population was restricted to commercially insured individuals, the patients ≥65 years of age captured in this study may represent a healthier population and our findings may not be generalizable to the elderly population insured through traditional fee-for-service Medicare. Furthermore, some patients ≥65 years with commercial insurance may be partially covered by traditional Medicare and their healthcare utilization data may be underestimated using this commercial claims database alone. These factors may be reflected in the relatively lower proportion of hospitalized influenza patients in our study ≥65 years (20.5%) compared to statistics reported by the CDC (50–70%) [28]. Despite this limitation, we anticipate that utilization would be evenly underestimated for both the patients with and without influenza and, thus, the impact on our findings evaluating the differences in outcomes between the matched groups would be limited.

## Conclusions

Overall, this real-world study reinforces previous research demonstrating a high healthcare resource burden attributable to influenza in elderly populations, but by varying degrees depending on their baseline comorbidity profile. Continued efforts to reduce influenza burden through prophylaxis are needed in high-risk populations in the US and globally.

## Supplementary Information

**Additional file 1: Supplementary File 1.** This file contains the ICD-9 and ICD-10 diagnosis codes used to identify high-risk comorbidity subgroups in Stage 2.

**Additional file 2: Supplementary Fig. 1.** Comparison of age and geographic distribution in IQVIA PharMetrics Plus compared to the 2019 US Census. **Supplementary Table 1.** Logistic regression model for the odds of influenza-related hospitalization, adjusting for baseline demographic and clinical characteristics. **Supplementary Table 2.** Samples sizes of the influenza and non-influenza cohorts before and after propensity score matching. **Supplementary Table 3.** Baseline demographic clinical characteristics for influenza and non-influenza patients with baseline asthma after PS-matching. **Supplementary Table 4.** Baseline demographic and clinical characteristics for influenza and non-influenza patients with baseline COPD after PS-matching. **Supplementary Table 5.** Baseline demographic and clinical characteristics for influenza and non-influenza patients with baseline chronic pulmonary disease after PS-matching. **Supplementary Table 6.** Baseline demographic and clinical characteristics for influenza and non-influenza patients with baseline atherosclerosis after PS-matching. **Supplementary Table 7.** Baseline demographic and clinical characteristics for influenza and non-influenza patients with baseline coronary artery disease (CAD) after PS-matching. **Supplementary Table 8.** Baseline demographic and clinical characteristics for influenza and non-influenza patients with baseline congestive heart failure (CHF) after PS-matching. **Supplementary Table 9.** Baseline demographic and clinical characteristics for influenza and non-influenza patients with baseline stroke after PS-matching. **Supplementary Table 10.** Baseline demographic and clinical characteristics for influenza and non-influenza patients with baseline valvular disease after PS-matching. **Supplementary Table 11.** Baseline demographic and clinical characteristics for influenza and non-influenza patients with history of old myocardial infarction (MI) after PS-matching. **Supplementary Table 12.** Baseline demographic and clinical characteristics for influenza and non-influenza patients with baseline acute MI after PS-matching. **Supplementary Table 13.** Baseline demographic and clinical characteristics for influenza and non-influenza patients with baseline early stage chronic kidney disease (CKD) after PS-matching. **Supplementary Table 14.** Baseline demographic and clinical characteristics for influenza and non-influenza patients with baseline late stage CKD after PS-matching.

## Data Availability

The data that support the findings of this study are available from IQVIA but restrictions apply to the availability of these data, which were used under license for the current study, and so are not publicly available. Data are however available from the authors upon reasonable request and with permission of IQVIA. Further details can be provided by contacting the A.N., the corresponding author.

## References

[1] Centers for Disease Control and Prevention (2020). Disease Burden of Influenza.

[2] Putri W (2018). Economic burden of seasonal influenza in the United States. Vaccine.

[3] Molinari NA (2007). The annual impact of seasonal influenza in the US: measuring disease burden and costs. Vaccine.

[4] Ghebrehewet S, MacPherson P, Ho A. Influenza. BMJ. 2016; 355:i6258. 10.1136/bmj.i6258. 10.1136/bmj.i6258 PMC5141587 27927672

[5] Tokars JI, Olsen SJ, Reed C (2018). Seasonal Incidence of Symptomatic Influenza in the United States. Clin Infect Dis.

[6] Centers for Disease Control and Prevention (2019). Influenza (Flu) Background and Epidemiology.

[7] Grohskopf LA (2017). Prevention and Control of Seasonal Influenza with Vaccines: Recommendations of the Advisory Committee on Immunization Practices - United States, 2017–18 Influenza Season. MMWR Recomm Rep.

[8] Wong KK (2015). Estimated paediatric mortality associated with influenza virus infections, United States, 2003–2010. Epidemiol Infect.

[9] Centers for Disease Control and Prevention (2021). People at Higher Risk of Flu Complications.

[10] Mertz D (2013). Populations at risk for severe or complicated influenza illness: systematic review and meta-analysis. BMJ.

[11] Van Kerkhove MD (2011). Risk factors for severe outcomes following 2009 influenza A (H1N1) infection: a global pooled analysis. PLoS Med.

[12] Near A, Tse J, Xu YY, Connolly L, Reyes C (2020). Incidence of medically-attended influenza and influenza-related hospitalisations by co-morbidities among a commercially insured population in the United States. European Congress of Clinical Microbiology and Infectious Diseases.

[13] Centers for Disease Control and Prevention (2020). Flu Vaccination Coverage, United States, 2019–20 Influenza Season.

[14] U.S. Census Bureau (2021). 2019 Population Estimates by Age, Sex, Race and Hispanic Origin.

[15] U.S. Census Bureau (2021). State Population Totals and Components of Change: 2010–2019.

[16] Cohen JP (2020). Estimation of the Incremental Cumulative Cost of HIV Compared with a Non-HIV Population. Pharmacoecon Open.

[17] Divino V (2020). A real-world study evaluating the relative vaccine effectiveness of a cell-based quadrivalent influenza vaccine compared to egg-based quadrivalent influenza vaccine in the US during the 2017–18 influenza season. Vaccine.

[18] Blumentals WA (2012). Rheumatoid arthritis and the incidence of influenza and influenza-related complications: a retrospective cohort study. BMC Musculoskelet Disord.

[19] Ehlken B (2015). Cost for physician-diagnosed influenza and influenza-like illnesses on primary care level in Germany--results of a database analysis from May 2010 to April 2012. BMC Public Health.

[20] Irwin DE (2001). Impact of patient characteristics on the risk of influenza/ILI-related complications. BMC Health Serv Res.

[21] Loughlin J (2003). A study of influenza and influenza-related complications among children in a large US health insurance plan database. Pharmacoeconomics.

[22] Rahmqvist M, Gjessing K, Faresjo T (2016). Influenza-related healthcare visits, hospital admissions, and direct medical costs for all children aged 2 to 17 years in a defined Swedish region, monitored for 7 years. Medicine (Baltimore).

[23] Uyeki TM, et al. Clinical Practice Guidelines by the Infectious Diseases Society of America: 2018 Update on Diagnosis, Treatment, Chemoprophylaxis, and Institutional Outbreak Management of Seasonal Influenza. Clin Infect Dis. 2019;68(6):e1–e47. 10.1093/cid/ciy866 PMC6653685 30566567

[24] Burudpakdee C (2019). 2758. Identifying Populations at High-Risk for Influenza-Related Hospitalization: A Real-World Analysis of Commercially Insured Population in the United States. Open Forum Infectious Diseases.

[25] Elixhauser A, Steiner C, Kruzikas D (2004). Comorbidity Software Documentation. HCUP Methods Series Report # 2004–1.

[26] Centers for Disease Control and Prevention (2018). People at High Risk of Developing Serious Flu–Related Complications.

[27] Stuart EA, Lee BK, Leacy FP (2013). Prognostic score-based balance measures can be a useful diagnostic for propensity score methods in comparative effectiveness research. J Clin Epidemiol.

[28] Centers for Disease Control and Prevention (2021). Flu & People 65 Years and Older.

[29] Beigel JH, Hayden FG. Influenza Therapeutics in Clinical Practice-Challenges and Recent Advances. Cold Spring Harb Perspect Med. 2021;11(4):a038463. 10.1101/cshperspect.a038463 PMC8015700 32041763

[30] Centers for Disease Control and Prevention (2021). Vaccine Effectiveness: How Well Do the Flu Vaccines Work?.

[31] US Department of Health and Human Services (2020). Immunization and infectious diseases: Objectives.

[32] Centers for Disease Control and Prevention (2021). Estimated Influenza Illnesses, Medical visits, Hospitalizations, and Deaths in the United States — 2019–2020 Influenza Season.

[33] CDC (2021). Estimated Influenza Illnesses, Medical visits, Hospitalizations, and Deaths in the United States — 2019–2020 Influenza Season.

[34] Kostova D (2013). Influenza Illness and Hospitalizations Averted by Influenza Vaccination in the United States, 2005–2011. PLoS One.

[35] Schmid P (2017). Barriers of Influenza Vaccination Intention and Behavior - A Systematic Review of Influenza Vaccine Hesitancy, 2005–2016. PLoS One.

[36] Coustasse A, Kimble C, Maxik K (2021). COVID-19 and Vaccine Hesitancy: A Challenge the United States Must Overcome. J Ambul Care Manage.

[37] Palache A (2015). Seasonal influenza vaccine dose distribution in 195 countries (2004–2013): Little progress in estimated global vaccination coverage. Vaccine.

[38] Crooke SN (2019). Immunosenescence and human vaccine immune responses. Immun Ageing.

[39] Centers for Disease Control and Prevention (2019). How CDC Estimates the Burden of Seasonal Influenza in the U.S.

