# Supplementary material for: Burden of influenza hospitalization among high-risk groups in the United States
Source: BMC Health Serv Res. 2022 Sep 28;22:1209. doi: 10.1186/s12913-022-08586-y (PMC9520810; doi:10.1186/s12913-022-08586-y)
Supplement: Supplementary file 1 — Additional file 1: Supplementary File 1. This file contains the ICD-9 and ICD-10 diagnosis codes used to identify high-risk comorbidity subgroups in Stage 2. [file 12913_2022_8586_MOESM1_ESM.docx]

**Supplementary File 1**

This file contains the ICD-9 and ICD-10 diagnosis codes used to identify high-risk comorbidity subgroups in Stage 2.

| Comorbidity | ICD-9 | ICD-10 |
| --- | --- | --- |
| Asthma | 49300, 49301, 49302, 49310, 49311, 49312, 49381, 49382, 49390, 49391, 49392 | J4520, J4521, J4522, J4530, J4531, J4532, J4540, J4541, J4542, J4550, J4551, J4552, J45901, J45902, J45909, J45990, J45991, J45998 |
| Chronic obstructive pulmonary disease (COPD) | 4910, 4911, 49120, 49121, 49122, 4918, 4919, 4920, 4928, 49320, 49321, 49322, 496 | J42, J430, J431, J432, J438, J439, J440, J441, J449 |
| Chronic pulmonary disease | 490, 494, 4940, 4941, 495, 4950, 4951, 4952, 4953, 4954, 4955, 4956, 4957, 4958, 4959, 500, 501, 502, 503, 504, 505, 5064, 51630, 51632, 51634, 51635, 51637, 5166, 51662, 51669, 5181, 7702, 507, 5070, 5078, 51636 | J40, J410, J411, J418, J470, J471, J479, J60, J61, J620, J628, J630, J631, J632, J633, J634, J635, J636, J64, J660, J661, J662, J668, J670, J671, J672, J673, J674, J675, J676, J677, J678, J679, J684, J703, J704, J84111, J84113, J84115, J84117, J8417, J842, J8484, J84842, J84848, J8489, J849, J982, P250, P258, J69, J690, J698, J84116 |
| Atherosclerosis | 440, 44023, 44029, 44324, 4403, 4409 | I70293, I70341, I7036, I7049, I70698, I70742, I70744, I70403, I70418, I7044, I70508, I70611, I70621, I7069, I7025, I70338, I70363, I70435, I70529, I70539, I70648, I70662, I70663, I70669, I70691, I70741, I70762, I70799, I70223, I70342, I70391, I7052, I70562, I70591, I70629, I70732, I70309, I70429, I7046, I70463, I7063, I707, I7074, I701, I70212, I70233, I70203, I70238, I70423, I70791, I708, I70232, I70218, I7022, I70263, I70519, I7056, I70602, I70643, I702, I70229, I70234, I70301, I70302, I70319, I70434, I70438, I70538, I7026, I70311, I70344, I70392, I70431, I70534, I706, I70632, I70339, I70399, I70441, I70593, I70609, I70631, I7071, I70292, I7033, I70332, I70398, I70468, I70541, I70668, I70328, I7035, I70201, I70323, I70411, I70461, I70513, I70521, I7054, I70618, I70644, I7065, I70701, I70798, I70419, I70708, I70542, I7060, I7020, I70243, I70493, I70544, I70711, I70719, I7075, I70211, I70244, I70329, I70401, I70499, I70511, I70512, I70532, I70641, I7076, I70245, I70303, I70549, I70563, I70213, I70222, I70239, I70312, I70428, I70469, I70518, I70569, I70722, I7091, I70219, I70262, I70361, I70318, I70622, I70412, I70433, I7045, I70633, I70409, I70522, I7061, I7066, I70723, I70733, I70748, I70749, I70413, I70502, I70592, I70634, I70709, I70738, I70763, I7021, I70343, I70345, I70368, I70393, I7050, I70568, I70608, I70703, I70721, I70739, I70792, I7090, I70269, I70298, I70362, I704, I70442, I70503, I70601, I70221, I70268, I70322, I70402, I70501, I7053, I70645, I70735, I70, I70228, I7023, I703, I70321, I70349, I70369, I70491, I7059, I7064, I7024, I70249, I70308, I70331, I70422, I7051, I70531, I70599, I70299, I7034, I70421, I70448, I70561, I70635, I70639, I7072, I70761, I70509, I70533, I7055, I70661, I7092, I70248, I7040, I70543, I70638, I70713, I70728, I70734, I7079, I70208, I70348, I7041, I70548, I70743, I70231, I70261, I70408, I705, I70619, I7062, I70702, I70712, I709, I70241, I70242, I70291, I7030, I70598, I70693, I70718, I7032, I70333, I70432, I70443, I70449, I70492, I70545, I7029, I7031, I7039, I7042, I70439, I70535, I70612, I70642, I70649, I70692, I70699, I70745, I70202, I70209, I70235, I70313, I70335, I7043, I70462, I70498, I7073, I70768, I70769, I70793, I70523, I70528, I70603, I7070, I70729, I700, I70334, I70444, I70445, I70613, I70623, I70628, I70731 |
| Coronary artery disease (CAD) | 414, 4140, 41400, 41401, 41402, 41403, 41404, 41405, 41406, 41407, 4141, 41410, 41411, 41412, 41419, 4142, 4143, 4144, 4148, 4149, 4292 | I25, I251, I2510, I2511, I25110, I25111, I25118, I25119, I253, I254, I2541, I2542, I255, I256, I257, I2570, I25700, I25701, I25708, I25709, I2571, I25710, I25711, I25718, I25719, I2572, I25720, I25721, I25728, I25729, I2573, I25730, I25731, I25738, I25739, I2575, I25750, I25751, I25758, I25759, I2576, I25760, I25761, I25768, I25769, I2579, I25790, I25791, I25798, I25799, I258, I2581, I25810, I25811, I25812, I2582, I2583, I2584, I2589, I259 |
| Congestive heart failure (CHF) | 39891, 428, 4280, 4281, 4282, 42820, 42821, 42822, 42823, 4283, 42830, 42831, 42832, 42833, 4284, 42840, 42841, 42842, 42843, 4289 | I0981, I501, I5020, I5021, I5022, I5023, I5030, I5031, I5032, I5033, I5040, I5041, I5042, I5043, I50810, I50811, I50812, I50813, I50814, I5082, I5083, I5084, I5089, I509 |
| Stroke | 43300, 43301, 43310, 43311, 43320, 43321, 43330, 43331, 43380, 43381, 43390, 43391, 43400, 43401, 43410, 43411, 43490, 43491, 4350, 4351, 4352, 4353, 4358, 4359, 436, 99702, V1254 | I6300, I63011, I63012, I63013, I63019, I6302, I63031, I63032, I63033, I63039, I6309, I6310, I63111, I63112, I63113, I63119, I6312, I63131, I63132, I63133, I63139, I6319, I6320, I63211, I63212, I63213, I63219, I6322, I63231, I63232, I63233, I63239, I6329, I6330, I63311, I63312, I63313, I63319, I63321, I63322, I63323, I63329, I63331, I63332, I63333, I63339, I63341, I63342, I63343, I63349, I6339, I6340, I63411, I63412, I63413, I63419, I63421, I63422, I63423, I63429, I63431, I63432, I63433, I63439, I63441, I63442, I63443, I63449, I6349, I6350, I63511, I63512, I63513, I63519, I63521, I63522, I63523, I63529, I63531, I63532, I63533, I63539, I63541, I63542, I63543, I63549, I6359, I636, I6381, I6389, I639, I6501, I6502, I6503, I6509, I651, I6521, I6522, I6523, I6529, I658, I659, I6601, I6602, I6603, I6609, I6611, I6612, I6613, I6619, I6621, I6622, I6623, I6629, I663, I668, I669, I97810, I97811, I97820, I97821, Z8673 |
| Valvular disease | 0932, 09320, 09321, 09322, 09323, 09324, 394, 3940, 3941, 3942, 3949, 395, 3950, 3951, 3952, 3959, 396, 3960, 3961, 3962, 3963, 3968, 3969, 397, 3970, 3971, 3979, 424, 4240, 4241, 4242, 4243, 4249, 42490, 42491, 42499, 7463, 7464, 7465, 7466, V422, V433 | A5203, I050, I051, I052, I058, I059, I060, I061, I062, I068, I069, I070, I071, I072, I078, I079, I080, I081, I082, I083, I088, I089, I091, I0989, I340, I341, I342, I348, I349, I350, I351, I352, I358, I359, I360, I361, I362, I368, I369, I370, I371, I372, I378, I379, I38, I39, Q230, Q231, Q232, Q233, Z952, Z953, Z954 |
| Old myocardial infarction (MI) | 412 | I252 |
| Acute MI | 41000, 41001, 41002, 41010, 41011, 41012, 41020, 41021, 41022, 41030, 41031, 41032, 41040, 41041, 41042, 41050, 41051, 41052, 41060, 41061, 41062, 41070, 41071, 41072, 41080, 41081, 41082, 41090, 41091, 41092 | I2101, I2102, I2109, I2111, I2119, I2121, I2129, I213, I214, I219, I21A1, I21A9, I220, I221, I222, I228, I229 |
| Early stage chronic kidney disease (CKD) | 5853, 5854, 5859, 5800, 5804, 58081, 58089, 5809, 5810, 5811, 5812, 5813, 58181, 58189, 5819, 5820, 5821, 5822, 5824, 58281, 58289, 5829, 5830, 5831, 5832, 5834, 5836, 5837, 58381, 58389, 5839, 7944 | N070, N071, N072, N073, N074, N075, N076, N077, N078, N079, N183, N184, N189, N000, N001, N002, N003, N004, N005, N006, N007, N008, N009, N010, N011, N012, N013, N014, N015, N016, N017, N018, N019, N020, N021, N022, N023, N024, N025, N026, N027, N028, N029, N030, N031, N032, N033, N034, N035, N036, N037, N038, N039, N040, N041, N042, N043, N044, N045, N046, N047, N048, N049, N050, N051, N052, N053, N054, N055, N056, N057, N058, N059, N060, N061, N062, N063, N064, N065, N066, N067, N068, N069, N08, R944 |
| Late stage CKD | 586, 5855, 5856, V420, V451, V4511, V4512, V56, V560, V561, V562, V563, V5631, V5632, V568 | N19, N185, N186, Z4901, Z4902, Z4931, Z4932, Z9115, Z940, Z992 |
